# Endoplasmic Reticulum Associated Protein Degradation (ERAD) in the Pathology of Diseases Related to TGFβ Signaling Pathway: Future Therapeutic Perspectives

**DOI:** 10.3389/fmolb.2020.575608

**Published:** 2020-10-29

**Authors:** Nesrin Gariballa, Bassam R. Ali

**Affiliations:** ^1^Department of Pathology, College of Medicine and Health Sciences, United Arab Emirates University, Al Ain, United Arab Emirates; ^2^Department of Genetics and Genomics, College of Medicine and Health Sciences, United Arab Emirates University, Al Ain, United Arab Emirates; ^3^Zayed Bin Sultan Center for Health Sciences, United Arab Emirates University, Al Ain, United Arab Emirates

**Keywords:** transforming growth factor, hereditary hemorrhagic telangiectasia, pulmonary arterial hypertension, ERAD, endoglin, BMPR2, ALK1

## Abstract

The transforming growth factor signaling pathway (TGFβ) controls a wide range of cellular activities in adulthood as well as during embryogenesis including cell growth, differentiation, apoptosis, immunological responses and other cellular functions. Therefore, germline mutations in components of the pathway have given rise to a heterogeneous spectrum of hereditary diseases with variable phenotypes associated with malformations in the cardiovascular, muscular and skeletal systems. Our extensive literature and database searches revealed 47 monogenic diseases associated with germline mutations in 24 out of 41 gene variant encoding for TGFβ components. Most of the TGFβ components are membrane or secretory proteins and they are therefore expected to pass through the endoplasmic reticulum (ER), where fidelity of proteins folding is stringently monitored via the ER quality control machineries. Elucidation of the molecular mechanisms of mutant proteins’ folding and trafficking showed the implication of ER associated protein degradation (ERAD) in the pathogenesis of some of the diseases. For example, hereditary hemorrhagic telangiectasia types 1 and 2 (HHT1 and HHT2) and familial pulmonary arterial hypertension (FPAH) associated with mutations in Endoglin, ALK1 and BMPR2 components of the signaling pathway, respectively, have all exhibited loss of function phenotype as a result of ER retention of some of their disease-causing variants. In some cases, this has led to premature protein degradation through the proteasomal pathway. We anticipate that ERAD will be involved in the mechanisms of other TGFβ signaling components and therefore warrants further research. In this review, we highlight advances in ER quality control mechanisms and their modulation as a potential therapeutic target in general with particular focus on prospect of their implementation in the treatment of monogenic diseases associated with TGFβ components including HHT1, HHT2, and PAH. In particular, we emphasis the need to establish disease mechanisms and to implement such novel approaches in modulating the molecular pathway of mutant TGFβ components in the quest for restoring protein folding and trafficking as a therapeutic approach.

## Introduction

**FIGURE 1:**
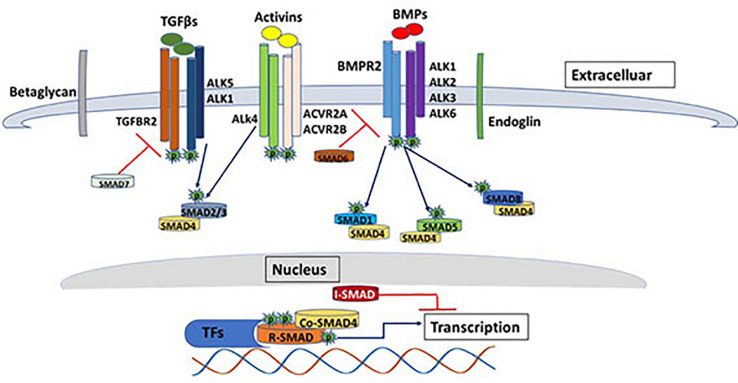
The TGF beta SMAD-dependent signaling pathway. The diagram shows some of the major components of the TGFβ signaling pathway. SMAD-dependents signal transduction is initiated with the binding of the ligands (e.g., TGFβ, BMP, activins etc.) to the serine/threonine type II receptor that phosphorylates and activates the type I receptor which causes dimerization of the type II and type I receptors in a heterotetrameric complex. The signal is then propagated to the nucleus through phosphorylation of SMAD transcription factors. The image represents only few of the ligands/receptors/SMADs possible signal transduction combinations.

Transforming growth factor (TGFβ) signaling pathway plays crucial roles in a diverse set of cellular activities such as cell growth, differentiation, immunological responses, apoptosis and during embryogenesis (Bobik, 2006; Caja et al., 2018; Goumans and Ten Dijke, 2018). The human genome encodes 42 of the TGFβ family protein ligands, which can be divided into two groups according to their sequence similarity and the pathways they activate (Lander et al., 2001). The first group includes the TGFβ, Activin and Nodal protein ligands while the second group includes bone morphogenetic protein (BMP), growth and differentiation factor (GDF), and Muellerian inhibiting substance (MIS) (Shi and Massagué, 2003). The signaling pathway is initiated via binding of the ligand to serine/threonine type II receptor that phosphorylates and activates the type I receptor which causes dimerization of the type II and type I receptors in a heterotetramic complex. The signal is then propagated to the nucleus through phosphorylation of SMAD transcription factors (Figure 1) (Shi and Massagué, 2003; Groppe et al., 2008).

In addition, the human genome encodes five type II receptors (Activin A Receptor Type 2A; ACVR2A, Activin A Receptor Type 2B; ACVR2B, Bone morphogenetic protein receptor; BMPR2, Activin A receptor like type 2; ACVRL2 and Anti-Müllerian hormone receptor;AMHR2) and seven type I receptors (Activin receptor-like kinase 1-7; ALK1-7) (Hata and Chen, 2016). Unlike type II receptors, type I receptors have a distinctive conserved 30 amino acid Glycine-Serine (GS) region (for the GSGS sequence it contains) that is phosphorylated by type II receptors (Huse et al., 1999). Type I and type II receptors are classified according to their sequence similarities. They are both dual specificity kinases due to having cytoplasmic kinase domain that has both serine/threonine kinase activity and tyrosine kinase activity (Gomez-Puerto et al., 2019). TGFβ ligands (TGFβ1, TGFβ2, and TGFβ3) are dimeric polypeptides that bind with high affinity to the TGFβ type two receptor, which phosphorylates the type I receptor, whereas, BMPs bind with equal affinity to both type I and type II TGFβ receptors (Massagué and Chen, 2000; Massagué, 2008; Kashima and Hata, 2018). TGFβ type III receptors (sometimes referred to as co-receptors) include Betaglycans and Endoglin, which both have no kinase activity, however they can bind all three TGFβ ligands with high affinity and facilitates ligand binding to TGFβ type II receptors and hence enhance ligand-receptor complex binding and augment its downstream signaling effect (Vander Ark et al., 2018).

SMADs are considered as the signal transducer in the TGFβ signaling pathway. They propagate the signal from cell membrane to the nucleus in a context dependence manner. Up to date, eight SMAD proteins have been identified in humans that have been classified into three classes, receptor mediated SMADs (R-SMADs), common partner SMADs (Co-SMADs) and inhibitory SMADs (I-SMADs) (de Caestecker, 2004). R-SMADs include SMAD2 and SMAD3 that are activated in response to binding of Activins and TGFβ proteins to the TGFBR2 and ACVR1B receptors. On the other hand, R-SMADs such as SMAD1, SMAD5 and SMAD8 are phosphorylated by BMPR2 receptor in response to the binding of BMP proteins. Activation of the second class SMADs (SMAD4-Co SMAD) is receptor independent, however their function is crucial for the receptor regulated SMADs. The third class of SMADs (SMAD6 and SMAD7) function as antagonists that inhibit the signaling of R-SMADs and Co-SMADs by competing with the ligands that trigger receptor phosphorylation. SMAD6 generally inhibits BMP activation, while SMAD7 generally work as a feedback regulator for TGFβ activation (Moustakas and Heldin, 2009; Pérez-Gómez et al., 2010; Hata and Chen, 2016). Beside the canonical SMAD regulated signaling pathway, TGFβ ligands can also regulate cellular physiological responses through non–SMAD signaling proteins, which have actually preceded the discovery of SMADs (Moustakas and Heldin, 2005). Non-SMAD signaling proteins downstream of the TGFβ receptors can attenuate and regulate the signaling pathways in various modes of actions. They can directly interact with type I receptors and become phosphorylated without a direct interaction with SMADs. On the other hand, non-SMAD proteins can transiently interact with SMADs in order to facilitate the activation of signaling pathways such as extracellular signal regulated kinase/mitogen-activated protein kinase (ERK/MAPK) pathways, Rho-like GTPase signaling pathways, and phosphatidylinositol-3-kinase (PI3K)/AKT pathways (Zhang, 2009). ERK/MAP kinase pathway is activated via tyrosine phosphorylation of ShcA by activated TGFβ type I receptor, followed by the formation of ShcA/Grb2/Sos complex and subsequent activation of Ras GTPase, Raf, MEK and ERK1/2 kinases. ERK1/2 can phosphorylate transcription factors as well as SMADs and hence regulate gene expression (Tzavlaki and Moustakas, 2020). The activation of the MAPK pathway is predominantly observed in epithelial cells triggered by a variety of cell growth stimuli such as insulin, thrombin, epidermal and hematopoietic growth factors (Hartsough and Mulder, 1995). c-Jun N-terminal kinase (JNK) and p38 MAP kinase pathways can also be activated independent of SMAD through the activation of MAP kinase kinases (MKKs). Both JNK and p38 MAP kinase pathways play key roles in a variety of cellular functions such as differentiation, apoptosis and inflammation (Yu et al., 2002). MAP3K7, also known as TGF-β-activated kinase 1 (TAK1), is a well-known activator of p38 MAP kinase pathway downstream of TGFβ ligands that can also phosphorylate R-SMADs at the linker region promoting a negative feedback regulation of the canonical TGFβ signaling pathway (Tzavlaki and Moustakas, 2020).

PIK3s exhibit constitutive interaction with type II receptors through its p85 regulatory unit, while interaction with the type I receptors occurs upon the TGFβ binding to the receptor complex, which leads to the activation of the PIK3/AKT signaling pathway (Yi et al., 2005). This pathway promotes cellular survival and growth in response to extracellular signals in multiple cellular processes including glucose metabolism, apoptosis and cell proliferation. Activation of PIK3 pathway can also activate mammalian target of rapamycin complex 2 (mTORC2), leading to the phosphorylation of AKT, which collectively contribute to epithelial–mesenchymal transition (EMT) and cell migration (Lamouille et al., 2012).

TGFβ ligands can also induce the Rho GTPases signaling pathway independent of SMADs regulation. Activation of RhoA and Cdc42 GTPases in epithelial cells play key roles in cytoskeleton regulation and cell motility (Edlund et al., 2002). This pathway can also be negatively regulated by Par6, a negative regulator of epithelial cells polarity that is closely associated with TGFβ type I receptor. Par6 phosphorylation facilitates the recruitment of ubiquitin ligases that labels RhoA GTPase for degradation (Ozdamar et al., 2005).

In addition to the SMAD and non-SMAD regulators of TGFβ signaling pathways, it is very important to note that cross-talk between the TGFβ signaling pathway and other pathways can also occur (Luo, 2017). The activation as well as the function of the various components of the TGFβ pathways are constantly regulated by various signaling pathways that control cellular processes, adding to the complexity and diversity of its functions.

## Involvement of TGFβ Signaling Pathway Components in Single Gene Disorders

As illustrated in the previous section, TGFβ signaling pathway plays a key role during the early embryonic developmental stages, in which axis formation and tissue specifications are determined (Harradine and Akhurst, 2006). Therefore, germline mutations in the TGFβ pathway components have given rise to a heterogeneous spectrum of hereditary diseases with phenotypes mainly associated with malformations in the cardiovascular, muscular and skeletal system. We have conducted an extensive literature and databases searches to document the involvement of mutations in the TGFβ pathway components in the development of monogenic hereditary diseases. This exercise revealed 47 monogenic diseases associated with genetic mutations in 24 out of 41 TGFβ components (Table 1). The majority of the diseases are autosomal dominant with variable penetrance and expressivity. Similar phenotypes can also arise from mutations affecting connected genes in the signaling pathway such as hereditary hemorrhagic telangiectasia type 1 and 2 (HHT1, HHT2), which are caused by mutations in ENG and *ACVRL1*, respectively. Our work and that of others have shown that the endoplasmic reticulum quality control (ERQC) pathways play a role in the pathogenesis of some of these diseases, which is the main focus of this manuscript.

**TABLE 1:** Monogenic diseases associated with TGFβ pathway components.

**Gene and Ensemble ID**	**Protein**	**Monogenic Disease/OMIM reference**	**Examples of pathological variants (Nucleotide/Amino acid change)**	**References**
ACVRL1 ENSG00000139567	Alk1	*(1) Hereditary hemorrhagic telangiectasia syndrome 2 (HHT2; 600376) (AD)	(1) c.1127T > G/p.M376R	Johnson et al., 1996
ACVR1 ENSG00000115170	Activin A receptor type 1 Alk2	(1) Fibrodysplasia ossificans progressiva (FOP; 135100) (A/D)	(1) c.617G > A/p.R206H	Shore et al., 2006
TGFBR1 ENSG00000106799	TGF beta receptor type1 Alk5	(1) Loeys-Dietz syndrome 1 (LDS1; 609192). (2) Multiple self-healing squamous epithelioma (MSSE; 132800)	(1) c.559C > T/p.T200I	Loeys et al., 2005 Ferguson-Smith et al., 1971
BMPR1A ENSG00000107779	Bone Morphogenetic Protein Receptor Type 1A Alk3	(1) Juvenile polyposis syndrome (JPS; 174900) (A/D). (2) Polyposis syndrome, mixed hereditary 2 (HMPS2; 610069) (A/D)	(1) c.715C > T/p. Q239Term (2) c.127_137del11/p.L43T(fs*24)	Howe et al., 2001 Cao et al., 2006
BMPR1B ENSG00000138696	Bone Morphogenetic Protein Receptor Type 1B Alk6	(1) Acromesomelic dysplasia, Demirhan type (AMDD; 609441) (A/R). (2) Brachydactyly A2 (BDA2; 112600) (A/D) (3) Brachydactyly A1, D (BDA1D; 616849) (A/D)	(1) c.361_368delGGACCTAT/p.G121T (fs*13) (2) c.1457G > A/p.R486Q (3) c.975A > C/p.K325N	Demirhan et al., 2005 Lehmann et al., 2003 Racacho et al., 2015
TGFBR2 ENSG00000163513	Transforming growth factor beta receptor 2	(1) Loeys-Dietz syndrome 2 (LDS2; 610168). (2) Hereditary non-polyposis colorectal cancer 6 (HNPCC6; 614331) (AD)	c.859T > C/p.W28R c.1063G > A/p. A355T	Boileau et al., 1993 Markowitz et al., 1995
BMPR2 ENSG00000204217	Bone Morphogenetic Protein Receptor Type 2	*(1) Pulmonary hypertension, primary, 1 (PPH1;17860) (AD) (2) Pulmonary venoocclusive disease 1; (PVOD1;265450) (AD)	(1) c.218C > G/p.S73Term. (2) c.727G > T/p.E243Term	Lane et al., 2000 Machado et al., 2001
ACVR2B ENSG00000114739	Activin A receptor type 2B	(1) Visceral heterotaxia4 (HTX4; 613751) (AR).	(1) c.119G > A/p.R40H	Kosaki et al., 1999
AMHR2 ENSG00000135409	Anti-Mullerian Hormone Receptor Type 2	(1) Persistent Muellerian duct syndrome type 2 (PMDS2; 261550) (AR)	(1) c.118G > T/p.G40Term	Imbeaud et al., 1995
ENG ENSG00000106991	Endoglin	*(1) Hereditary hemorrhagic telangiectasia syndrome 1 (HHT1; 187300) (AD)	(1) (Trp196Term/c.587G > A)	McAllister et al., 1994
BMP1 ENSG00000168487	Bone Morphogenetic Protein 1	(1) Osteogenesis imperfecta 13 (OI13; 614856)	(1) c.747C > G/p.F249L	Martínez-Glez et al., 2012
BMP2 ENSG00000125845	Bone Morphogenetic Protein 2	(1) Brachydactyly A2 (BDA2;112600) (A/D) (2) Short stature, facial dysmorphism, and skeletal anomalies with or without cardiac anomalies (SSFSC; 617877) (A/D)	(1) (Duplication)5547 bp, ∼110 kb downstream of gene (2) c.79G > T/p.E27Term	Dathe et al., 2009 Tan et al., 2017
BMP4 ENSG00000125378	Bone Morphogenetic Protein 4	(1) Microphthalmia, Syndromic 6 (A/D) (MCOPS6; 607932) (2) orofacial cleft 11 (OFC11; 600625)	(1) c.278A > G/p.E93G (2) c.485G > A/p.R162Q	Bakrania et al., 2008 Suzuki et al., 2009
BMP9/GDF2 ENSG00000263761	Morphogenetic Protein9/Growth Differentiation Factor 2	(1) Hereditary hemorrhagic telangiectasia type5 (HHT5; 615506) (AD)	c.76C > T/p.q26Term.	Wang et al., 2016
GDF1 ENSG00000130283	Growth Differentiation Factor 1	(1) Congenital heart defects, multiple types, 6 (CHTD6; 613854) (AD). (2) Right atrial isomerism (RAI; 208530)	(1) c.203G > A/p.R68H (2) c.1322T > C/L441P	Karkera et al., 2007 Eronen et al., 2004
GDF3 ENSG00000184344	Growth Differentiation Factor 3	(1) Klippel-feil syndrome 3 (KFS3; 613702) (AD). (2) Microphthalmia, isolated, 7 (MCOP7; 613704) (AD). (3) Microphthalmia, isolated, with Coloboma 6 (MCOPCB6; 613703) (AD).	(1) c.796C > T/p.R266C (2) c.914T > C/p.R195Q (3) c820C > T/p R274W	Ye et al., 2010
GDF5 ENSG00000125965	Growth Differentiation Factor 5	(1) Acromesomelic chondrodysplasia Hunter-Thomson type (AMDH;201250) (AR). (2) Acromesomelic chondrodysplasia, Grebe type (AMDG; 200700) (AR) (3) Brachydactyly C (BDC;113100) (AD and AR) (4) Du Pan syndrome (DUPANS; 228900) (AR) (5) Symphalangism, proximal 1B (SYM1B; 615298) (6) Multiple synostoses syndrome 2 (SYNS2; 610017). (7) Brachydactyly A2 (BDA2; 112600). (8) Osteoarthritis 5 (OS5; 612400) (9) Brachydactyly A1, C (BDA1C; 615072)	(1/2) c.1199G > A/p.C400Y (3) c.122delG/p.(Gly41Aspfs*46) (4) c.1322T > C/p.L441P (5) c.1313G > T/p.R438L (7) c.1139G > A/p.R380Q (8) c.-275C > T (Regulatory) (9) c.1195C > T/p.R399C	Thomas et al., 1997 Polinkovsky et al., 1997 Faiyaz-Ul-Haque et al., 2002 Seemann et al., 2005 Plöger et al., 2008 [Bibr B98][Bibr B15]
TGFb1 ENSG00000105329	Transforming Growth Factor Beta 1	(1) Camurati-Engelmann disease (CAEND; 131300) (2) Inflammatory bowel disease, immunodeficiency and encephalopathy (IBDIMDE; 618213)	(1) c.652C > T/p.R218C (2) c.328C > T/p.R110C	Kinoshita et al., 2000 Kotlarz et al., 2018
TGFb2 ENSG00000092969	Transforming Growth Factor Beta2	(1) Loeys-Dietz syndrome 4 LDS4; 614816 (AD)	(1) c.297C > A/p.Y99Term.	Lindsay et al., 2012
TGFb3 ENSG00000119699	Transforming Growth Factor Beta	(1) Loeys-Dietz syndrome 4 (LDS5; 615582) (AD) (2) Arrhythmogenic right ventricular dysplasia-1 (ARVD1; 107970)	(1) c.1226G > A/p.C409Y (2) c.-30G > A (Regulatory)	Rienhoff et al., 2013 Rampazzo et al., 2003; Beffagna et al., 2005
SMAD3 ENSG00000166949	SMAD Family Member	(1) Loeys-Dietz syndrome 3 (LDS3; 613795) (AD)	(1) c.782C > T/p.T261I	van de Laar et al., 2011
SMAD4 ENSG00000141646	SMAD Family Member	(1) Juvenile polyposis syndrome/hereditary hemorrhagic telangiectasia syndrome (JPS; 174900) (AD) (2) (JP/HHT) (JPHT, 175050) (AD), (3) Myhre syndrome (MYHRS; 139210)	(1) c.1042_1043delGT/p.(Val348Tyrfs*3) (2) c.1157G > A/p.G386D (3) c.1500A > G/p.I500M	Howe et al., 1998 Burger et al., 2002 Le Goff et al., 2011
SMAD6 ENSG00000137834	SMAD Family Member	(1) Aortic valve disease (AOVD2; 614823) (2) Craniosynostosis (CRS7; 617439) (3) Radioulnar synostosis (RUS; 179300)	(1) c.1244C > T/p.P415L (2) c.968C > T/p.P323L (3) c.461G > A/p.G154D	Tan et al., 2012 Timberlake et al., 2016 Yang et al., 2019
SMAD8/9 ENSG00000120693	SMAD Family Member	(1) Primary pulmonary hypertension 2 (PPH2; 615342)	(1) c.606C > A/p.C202*	Shintani et al., 2009

The table lists all SMAD-regulated components of the TGFβ signaling pathway and associated monogenic diseases. Components with no identified association are (ACVR1B, ACVR1C, ACVR2A, TGFBR3, INHBA, Inhibin subunits A, B, BB and C, BMP 3, 5, 6, 7 and 10, GDF7, SMAD 1, 2, 5 and 7). Genetic diseases found to have a possible ERAD pathology are denoted with an asterisk*. AR, autosomal recessive; AD, autosomal dominant.

## ERAD Components and Mechanisms

**FIGURE 2:**
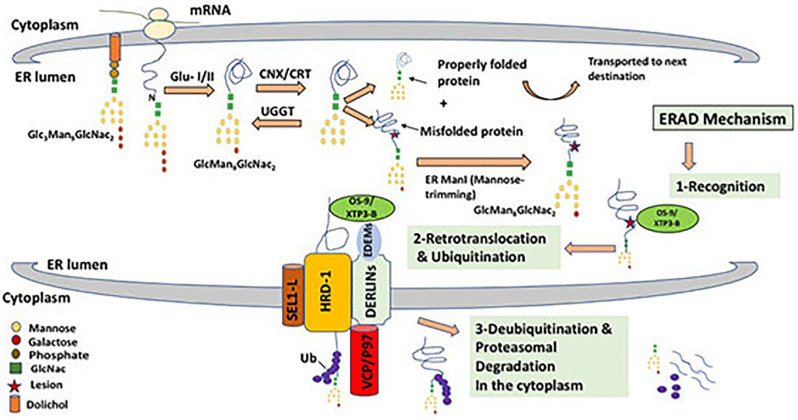
ERAD mechanism for misfolded glycoprotein through the HRD-1/SEL-1L complex. Triglycosylated form of protein-bound oligosaccharide (Gl_3_Man_9_GlcNac_2_) is processed by glucosidase enzymes (GluI/II) that cleaves off two glucose molecules. This is followed by cycles of interaction between the nascent protein and lectins such as Calnexin and Calreticulin (CNX/CRT), that binds specifically to monoglucosylated oligosaccharides (GlMan_9_GlcNac_2_) and ensure the proper folding of newly synthesized protein. This cycling effect is generated by the enzyme UDP-glucose:glycoprotein glucosyltransferase (UGGT), that transfers back a glucose residue to the improperly folded protein to enable further encounters with the ER chaperones (CNX/CRT). A properly folded protein is then released after the cleavage of the remaining glucose molecule. Properly formed protein is dispatched to its functional destination, while misfolded protein that cannot reach its mature form will undergo mannose cleavage by ER α1,2-mannosidase I (ERMan1), which produces Man8GlcNAc2. Terminal mannose cleavage (α mannose) function as a recognition signal for ERAD lectins OS-9 and XTP3-B that recognize and binds to exposed mannose residues after cleavage of α mannose. The three chaperones EDEM1, OS9 and XTP3-B function together as recognition complex that interacts with misfolded proteins and the HRD-1/SEL-1L retrotranslocation channel. Derlins which are candidates for the translocon channel also interacts with the EDEMs and facilitates the interaction of EDEMs with cytosolic AAA-ATPase p97, that provides ATP hydrolysis for successful extraction of mutant proteins. Retrotranslocation is coupled by Uniquitination, a process that targets proteins for degradation by 26S proteasome by tagging them with ubiquitin chains.

The Endoplasmic Reticulum (ER) has adopted a highly sophisticated, stringent and conserved quality control mechanism known as ER-associated protein degradation (ERAD) to dispose of improperly folded secretory and membrane proteins and orphaned subunits of protein complexes (Preston and Brodsky, 2017; Sun and Brodsky, 2019). ERAD is a complex process that involves the coordination of the functions of many proteins in both the ER and the cytoplasm with input from the nucleus through the unfolded protein response (UPR); a cellular adaptive mechanism to resolve ER stress (Christianson and Ye, 2014). In order for a misfolded protein to be discarded, it needs to be recognized, retrotranslocated into the cytosol, polyubiquitinated and then extracted from the ER membrane to be degraded in the cytosol by the ubiquitin/proteasomal system (Figure 2) (Wu and Rapoport, 2018).

Newly synthesized proteins that successfully attain native conformations and assemble into complexes, if needed, with the assistance of resident ER molecular chaperones are usually allowed to be incorporated into vesicles and transported further to reach their final destinations and organelles within the secretory pathway or be secreted outside the cell (Needham and Brodsky, 2013). However, fully or partially misfolded proteins and orphaned proteins are retained in the ER and then usually get degraded by the ubiquitin proteasomal systems (Sun and Brodsky, 2019). On the other hand, it became clear recently that some misfolded proteins aggregate in the secretory pathway and are targeted and degraded by autophagy (Qi et al., 2017). Therefore, recently, the umbrella term ERALD (ER-to-lysosomes-associated degradation) for autophagic and non-autophagic pathways triggered by ERAD-resistant misfolded proteins, has been proposed (Fregno and Molinari, 2019). Figure 2 shows the possible fates and stages of ER misfolded proteins. Proteins that are structurally mutated are likely to fail reaching the proper conformation and will possibly be transiently trapped in the ER. The length of stay in the ER before the decision to degrade the misfolded or malfolded protein varies from one protein to another. Accumulation of misfolded proteins in the ER is often cytotoxic and may cause ER stress leading to an array of metabolic, immune and degenerative diseases (Sha et al., 2011; Hetz, 2012; Zito, 2019). In addition, unresolved mutated proteins may form aggregates that disrupt ER homeostasis and trigger UPR that activates expression of molecular chaperones that can process aberrant proteins as well as transcription factors that attenuate protein translation to reduce protein synthesis load on the ER (Nishikawa et al., 2005). The first and perhaps the most crucial step in ERAD is the recognition of an ERAD substrate in the highly crowded ER environment that harbors a whole spectrum of newly synthesized proteins in varying stages of their folding, oligomerization and post-translation modifications. Therefore, commitment to degrade a particular protein through ERAD is highly specific and must, therefore, be very tightly controlled. In order to differentiate between permanently misfolded and partially folded native proteins, both protein species are kept in their soluble form bound to the mammalian ER molecular chaperone Hsp70 (BiP in mammals). BiP recognizes the exposed hydrophobic regions of improperly folded protein species and plays a key role in folding, or otherwise, disposal through the ERAD pathway due to the prolonged association with BiP (Stevenson et al., 2016). On the other hand, N-linked glycoproteins have a characteristic and well-defined glycan moiety of three glucose, nine mannose, and two *N*-acetylglucosamine residues (Glc3–Man9–GlcNAc2). Glucosidases (GLU I and II) catalyze the cleavage of two glucose residue from a triglycosylated form of protein-bound oligosaccharide and hence facilitate the binding of ER molecular chaperones to monoglycosylated glycoproteins (GlMan_9_GlcNac_2_) which in turn play a crucial role in glycoprotein folding and processing (Ruggiano et al., 2014; Tax et al., 2019). Glycoprotein lectins such as calnexin and calreticulin (CNX/CRT) facilitate protein folding through repeated cycles of interaction with nascent proteins via their high binding affinity to the mono-glucose residue. However, if the protein fails to fold properly these two chaperones recognize specific N-linked carbohydrate moiety structures that are associated with glycoproteins and target them for ERAD. Terminally misfolded glycoproteins are extracted from CNX/CRT by members of the EDEM (ER degradation-enhancing α-mannosidase-like protein) family (EDEM1-3) and ER mannosidase I. Mannosidase I removes one mannose residue from the glycoprotein in a process known as “mannose trimming” that facilitates substrate transfer from calnexin to EDEM, a step that signals initiation of the ERAD mechanism (Oda et al., 2003; Nishikawa et al., 2005). OS-9 and XTP3-B are other ER resident lectins that bind luminal misfolded glycol proteins to the Hrd1 E3 ligase through the transmembrane adaptor protein (SEL-1L) (Christianson et al., 2008). Once a mutant protein is recognized and selected as a substrate for disposal by ERAD, it is transferred out of the ER to the cytoplasm for degradation through a process referred to as retrotranslocation or dislocation. Despite the fact that the mechanisms by which a mutant protein is channeled through the ER membrane to the cytosol is still not clear, it has been established that E3 ubiquitin ligases coordinate the execution of this crucial step (Ruggiano et al., 2014). The best-characterized E3 ligases in mammalian cells are Hrd1 and GP78, which are both ER multi spanning membrane proteins with a RING domain responsible for the ligase activity in the cytoplasm (Bernasconi et al., 2010; Joshi et al., 2017). In yeast, it has been characterized that E3 ligase complexes are specific to the location of the lesion (mutation) on the ERAD substrate. For example proteins with cytoplasmic lesions (ERAD-C) are degraded through the Doa10 E3 ligase complex, whereas proteins with structural misfolding in their luminal (ERAD-L) or intramembrane (ERAD-M) are degraded through the Hrd1 E3 ligase complex (Vashist and Ng, 2004). Asi E3 ligase complex has recently been identified and its substrates included soluble and intramembrane proteins (Foresti et al., 2014; Stevenson et al., 2016). In mammalians, correlation between the type of ERAD substrate and choice for degradation pathway has also been identified in few cases, however it is still not clear what dictates a particular pathway for mammalian ERAD substrates and that is primarily due to the complexity of the retrotranslocation channels and its associate proteins (Lemus and Goder, 2014). Unlike in yeast, several mammalian E3 ligases, beside Hrd1 and GP78, have been implicated in the ERAD substrate dislocation such as RMA1, TEB4, RFP2, TRC8, Kf-1, RNF170 and Nixin/ZNRF4 (Claessen et al., 2012). Other ER trans-membranous proteins such as Derlin-1, Derlin-2 and Derlin-3 (Der1p family) also associate with the E3 ligases and other ERAD factors to form a complex retrotranslocation channel spanning the ER membrane. In mammals, Derlins play a major role in the retrotranslocation of luminal substrates, however their exact function remains speculative (Huang et al., 2013; Christianson and Ye, 2014).

It has been identified that ERAD substrate dislocation is driven by a cascade of ubiquitination enzymatic activities that labels defective proteins that should undergo proteasomal degradation with a chain of four or more ubiquitins attached to lysine 48 to be recognized by the 19S cap of 26S proteasome (Preston and Brodsky, 2017). Ubiquitin ligase enzyme (E1) activates a ubiquitin-conjugating enzyme (E2), of which there are 40 enzymes. They together work in conjunction with a ubiquitin ligase (E3), of which there are 650 enzymes, to transfer ubiquitin to the selected ERAD substrate (Christianson and Ye, 2014; Caldeira et al., 2014). The attachment of a poly-ubiquitin chain to ERAD substrates triggers the recruitment of AAA + ATPase Cdc48 (p97/VCP in mammals) that provides the energy for the extraction of nearly all EAD substrates (Stolz et al., 2011). However, the fact that p97/VCP is a cytoplasmic protein means it can only interact with luminal ERAD substrates after they are partially out of the ERAD translocon channel, which raises many questions about the origin of the energy that drives the initiation of the retrotranslocation process (Olzmann et al., 2013). Mammalian E2s such as UBE2J1, UBE2J2, and UBE2G2 have also been implicated in the dislocation process, however their specificities toward a particular E3 ligase have not yet been established (Claessen et al., 2012). Ubiquitination in ERAD is also controlled by the opposing effect of deubiquitinases (DUB) that remove ubiquitin chains conjugated with ERAD substrates. DUBs were found to be in close association with p97/VCP and 26S proteasome, which suggests their role in substrate dislocation and proteasomal function. It has been proposed that this association facilitates the threading of unfolded proteins to be degraded in the proteolytic chamber of 26S proteasome (Pickart and Cohen, 2004; Ernst et al., 2011).

## Involvement of ERAD in Disease Mechanisms

The ERAD quality control mechanism has been involved in the pathogenesis of numerous genetic conditions including cystic fibrosis, emphysema and Robinow syndrome and Alzheimer Diseases (AD), Parkinson disease and other neuro degenerative diseases (Reviewed in Kaneko et al., 2017). This is simply because almost a third of all the cellular proteins need to be targeted to the ER in transit to their final destinations and are therefore subjected to this highly stringent quality control system (Sun and Brodsky, 2019). On one hand, failure of ERAD mechanism to degrade accumulated mutant proteins induces ER stress which often leads to cellular toxicity and possibly cell death. On the other hand, degradation of a mutant, but functional, protein is also likely to deprive the cells of an important functional protein leading to loss of function phenotypes which have been observed for numerous hereditary diseases. HRD1 E3 ligase has been shown to be involved in the elimination of amyloid precursor protein (APP) and therefore prevents its accumulation (Kaneko, 2016). Reduced levels of HRD1 has been observed in cerebral cortex tissues of patients with AD, which implicates the ERAD in the pathogenesis of this disease (Kaneko et al., 2002). Parkin (*PARK2*), is another ERAD ubiquitin ligase that is involved in the pathogenesis of familial Parkinson diseases (PD) (Ishikawa and Tsuji, 1996). Mutations in *PARK2* leads to accumulation of its substrate in the ER leading to ER stress-induced neuronal cells death (Imai et al., 2001). Mutant proteins have also been shown to interfere with ERAD components impairing their functionality in a number of neurodegenerative diseases such as ALS and Huntington disease (Nishitoh et al., 2002, 2008).

## HHT1, HHT2, and FPAH: Angiogenic Molecular Pathology and Current Implication of ERAD Mechanism

Hereditary hemorrhagic telangiectasia types 1 and 2 (HHT1, HHT2) and familial pulmonary arterial hypertension (FPAH) have been associated with mutations in *ENG, ACVRL* and *BMPR2*, respectively. The mutant proteins encoded by the three genes (Endoglin, Alk1 and BMPR2) are transmembrane receptors in the TGFβ signaling superfamily (McAllister et al., 1994; Johnson et al., 1996; Lane et al., 2000).

### Endoglin and ALK1

Endoglin is a homodimeric transmembrane protein that acts as a TGFβ co-receptor; encoded by the gene *ENG* on chromosome 9q33-q34.1. It binds with high affinity to TGFβ ligand as well as BMP9 and BMP10 in the presence of Alk1 and TGFBR2 which ultimately leads to the activation of SMAD 1/5/8 transcription factors that enter the nucleus leading to upregulation of genes that promote endothelial cells angiogenesis (Guerrero-Esteo et al., 2002; Castonguay et al., 2011; Ollauri-Ibáñez et al., 2017). Endoglin is predominantly expressed in vascular endothelium of the heart, liver and brain and it is therefore essential for the normal structure of the vascular system in humans (Castonguay et al., 2011). Mutations in *ENG* result in the vascular disease hereditary hemorrhagic telangiectasia type1 (HHT; OMIM 187300), also known as Osler-Rendu-Weber syndrome 1, an autosomal dominant vascular syndrome (McAllister et al., 1994). The disease affects 1 in 5000–8000 people, however evidence of the disease may not be present until the age of 30 (Abdalla and Letarte, 2006). Early manifestation of the disease can present in recurrence of nasal bleeds (epistaxis) that may require blood transfusion. The complexity of the disease arises from its variable phenotypic nature, however it is usually presented in adults with either large arteriovenous malformation (AVM) present in the lungs, liver, gastrointestinal tract, and brain or small cutaneous and mucous membrane telangiectases (Richards-Yutz et al., 2010). Telangiectases can develop on the face, lips, fingers, mouth and gastrointestinal tracts leading to hemorrhage and anemia, on the other hand, AVM accounts for devastating consequences such as stroke, fatal hemorrhage and heart failure (Karabegovic et al., 2004).

Hereditary hemorrhagic telangiectasia type 2 (HHT2; OMIM 600376) is caused by mutations in Activin receptor-like kinase gene (*ACVRL1*) in chromosome 12q13 (Johnson et al., 1996). The gene encodes a type one receptor in the TGFβ pathway;ALK1. Upon ligands BMP9 and BMP10 binding, a receptor complex of two type II and two type I transmembrane serine/threonine kinase is formed. Activated ALK1 signals a particular transcriptional response through the SMADs transduction pathway (Mehnert et al., 2010; Alaa El Din et al., 2015). Endoglin was shown to enhance BMP9-induced ALK1 signaling pathway in endothelial cells (Nolan-Stevaux et al., 2012). Mutations in *ENG* and *ACVRL1* genes disrupts recruitment of pericytes, that stabilizes endothelial cells during capillary development, leading to capillary malformation in both HHT1 and HTT2 syndromes (Vorselaars et al., 2018). Both HHT1 and HHT2 have similar phenotype and mutations in both genes account for nearly 85% of all HHT cases while the remaining cases are associated with mutations in SMAD4 or other unknown genes (Gallione et al., 2004). Mutation in the SMAD4 gene actually cause combination of Juvenile polyposis and HHT and only account for 2% of all HHT cases (McDonald et al., 2011).

TGFβ signaling pathway has been implicated in the range of cardiovascular diseases due to the key role of the TGFβ ligand secreted by endothelial cells in stabilizing mature vascular network, reviewed in Goumans and Ten Dijke (2018). Although vasculogenesis is established during embryogenesis, formation of new blood vessels for growing tissues and during wound healing will continue all through an individual’s life. Angiogenesis is regulated by a variety of cytokines and growth factors such as TGFβ and vascular endothelial growth factor VEGF (Ferrari et al., 2009). TGFβ1 is a potent proliferation inhibitor and apoptotic inducer in various cell types (Massagué et al., 2000). This key feature facilitates the angiogenic effect of the ligand as apoptosis is required for pruning of the newly formed vessels. Inhibition of the pathway due to genetic mutations or any other external factors can inhibit the apoptotic effect and result in the formation of abnormal vascular network. During angiogenesis VEGF induces endothelial cells proliferation and migration to the extracellular space where new vessels are formed. This stage is followed by a maturation stage when endothelial cells secrete TGFβ to recruit mesenchymal cells to be differentiated to pericytes and smooth muscle cells (SMCs) that stabilize the newly formed vessel (Carmeliet and Jain, 2011). In the final stages of vascular developments, VEGF steer vascular remodeling and pruning of non-functional sprouts that fail to model in a way that suites that particular tissue needs (Armulik et al., 2011). The process of vessel formation is tightly controlled by angiogenic as well as angiostatic factors that halt the angiogenesis in a context dependent manner. However, in pathological conditions such as HHT, angiogenesis lacks the natural fine tuning, which leads to persistent and excessive formation of abnormal vessels such as AVMs. Several animal and clinical studies have implicated angiogenic VEGF in the pathology of HHT, which lead to the proposal of anti VEGF therapies for the management of HHT (Sadick et al., 2005; Shao et al., 2009; Choi et al., 2014). Systematic treatment of HHT patients with the humanized anti-VEGF antibody (bevacizumab, 5–10 mg/kg) have shown improvement in the frequency of epistaxis, the number of required transfusion and improved liver function in patients with severe phenotype (Ardelean and Letarte, 2015). Despite the promising prospect of this therapy, management of side effects such as gastrointestinal bleeding, proteinuria and hypertension remain to be an obstacle (Pavlidis and Pavlidis, 2013). Moreover, the mechanism of action by which bevacizumab exerts theses effects is poorly understood, and also its effects on AVMs in the brain and lungs has not yet been studied. Thalidomide is another drug with anti-angiogenic and immunomodulatory properties that have been tested on HHT. Administration of thalidomide to a group of HHT patients have reduced the frequency of epistaxis, blood transfusion and GI bleeding, however, the thrombogenic nature of the drug have caused side effects including peripheral neuropathy and deep vein thrombosis (Franchini et al., 2013). On the other hand, second-generation analog of thalidomide (lenalidomide) have shown a better safety profile, in addition to its anti-angiogenic properties via the direct inhibition of VEGF production and endothelial cell migration (Teo, 2005). Recently, thalidomide and lenalidomide were also shown to improve mural cell coverage of brain AVMs in mouse model and hence reduce incidence of brain hemorrhage (Zhu et al., 2018). Since then, a lot of research has been carried out in order to improve the efficacy and safety profile of such innovative therapies. However, in recent years the focus has shifted to the molecular pathway through which TGFβ secretory proteins are transported. The secretory pathway is a complex network of vesicular compartment through which proteins and cellular components are transported from the ER to the Golgi apparatus to the extracellular space, recycled back to the endosome and lysosome (Mercado et al., 2016). Genetic mutations of secretory proteins can lead to their aggregation in the ER lumen and premature degradation through ERAD, a mechanism that is most likely to be implicated in the pathology of HHT type 1 and 2 (Ali et al., 2011; Hume et al., 2013).

Over 400 mutations in *ENG* have been reported to cause HHT1, however the underlying mechanisms of the disease have not been thoroughly investigated (Mallet et al., 2015). In 2011, we examined the subcellular trafficking of 28 disease-causing missense mutations in *ENG*. Subcellular localization of wild type and mutant variants of *ENG* has been examined using both confocal microscope and analysis of their *N*-glycosylation profiles. The results revealed that 10 out of 28 mutants have localized in the ER rather than the endogenous localization in the plasma membrane (Ali et al., 2011). These findings gave an evidence that, in some patients, defective trafficking of endoglin from the ER is the most likely mechanism underlying HHT1.

Functional assays developed to investigate the pathogenicity of Endoglin and ACVRL1 mutations have suggested variable mechanisms for their loss of function including their retention intracellularly in the ER (Ricard et al., 2010; Mallet et al., 2015). We have also shown in a previous study the implication of ERAD mechanism in the pathogenesis of HHT2 caused by *ACVRL1* mutations (Hume et al., 2013). Wild-type ALK1 and a number of HHT2 patient mutant variants were expressed as C-terminally tagged EGFP fusion proteins and their localization was tested in HeLa cells. Wild type ALK1 was found to be predominantly targeted to the plasma membrane, where it executes its function as a type 1 TGFβ receptor. On the other hand, the majority of overexpressed mutant ALK1 proteins were found to be retained in the ER. It was therefore reasonable to predict that defective trafficking of mutant ALK1 receptors, followed by premature degradation through ERAD mechanism are possible mechanism for HHT2 in some patients (Hume et al., 2013).

### BMPR2 (Bone Morphogenetic Protein Receptor Type 2)

BMPR2 encodes for a type II receptor in the TGFβ signaling superfamily. Mutations in this gene have been identified in 80% of families with multiple cases of pulmonary arterial hypertension (PAH) (Lane et al., 2000). The remaining 20% of families affected by the disease are recognized as sporadic; as they have no detectable mutations in currently known disease-associated genes. In addition, 5% of PAH patients are actually patients of HHT1 and HHT2 diseases (Simonneau et al., 2013). PAH is generally characterized by elevated arterial pressure caused by abnormal proliferation of endothelial cells of the arteries, which eventually leads to heart failure and death. The disease has similar manifestations in its Familial (FPAH), Idiopathic (IPAH) or sporadic form. Structural changes in the pulmonary vasculature is the main feature of PAH. BMP4 and BMP6 induce vascular endothelial cell proliferation and migration, while BMP9, which is a specific ligand to both BMPR2 and ALK1, was shown to inhibit excessive proliferation and protects endothelial cells from apoptosis (Morrell et al., 2019). This finding may explain the occurrence of PAH diseases in families affected with HHT2 due to mutated ALK1 protein.

There are four distinct functional domains in the mature BMPR2 protein, consisting of extra cellular ligand binding domain, a transmembrane region, a serine/threonine kinase domain (KD), and a cytoplasmic tail domain. The majority of the diseases causing mutation mutations encoding region of BMPR2 are frameshift, nonsense mutation or deletions that triggers mutant mRNA decay through the mechanism nonsense mediated decay (NMD) (Machado et al., 2009). The rest of BMPR2 mutations identified in PAH patients are missense mutations affecting functional domain in the receptor (Li et al., 2010).

Sub-cellular localization of some missense mutant BMPR2 protein in the ligand binding domain affecting highly conserved cysteine residues resulted in retention of the mutant proteins in the ER, whereas mutation in the kinase domains showed localization to both ER and plasma membrane. On the other hand, cytoplasmic tail mutants localized exclusively in the plasma membrane (John et al., 2015). These findings consolidate the implication of the ER quality control mechanism in the pathogenesis of FPAH. A study by Satow et al. (2006) has also shown that BMPR2 receptor is degraded via the proteasomal pathway in a negative feedback mechanism promoted by Dullard; a gene involved in neural development in xenopus. Recently, autophagy has also been shown to contribute to the degradation process of BMPR2 receptor in primary human pulmonary artery endothelial cells (PAECs) (Gomez-Puerto et al., 2019). Discrepancy in the results between the two studies probably suggest more than one degradation pathway for the BMPR2 receptor depending on the nature of the mutation and cell type.

Up until now, conventional therapies for PAH including prostaglandins, phosphodiesterase-5 inhibitors, endothelin receptor antagonists, and soluble guanylate cyclase stimulators aim to improve functional capacity and reduce hospital admissions. However, these vasodilators have not been successful in reversing the disease pathology or reducing the disease mortality rate (Thenappan et al., 2018). Current therapeutic approach for the treatment of PAH have recently focused on restoring the BMP signaling pathway by using the immunosuppressive drug (tacrolimus). The drug was shown to ameliorate the symptoms in PAH patients and prevent the development of PAH in BMPR2 deficient mice (Spiekerkoetter et al., 2015; Sommer et al., 2019). Therefore, we predict that functional restoration of defective protein trafficking through ERQC manipulation will open new windows for such innovative therapeutic approaches.

## Manipulation of the ER Quality Control as a Therapeutic Target

Involvement of the ERAD mechanism in the pathogenesis of these diseases potentially opens a window for novel therapeutic targets which involves the manipulation of the ER quality control mechanism to enhance mutant protein folding and trafficking. The quality control of protein-folding in the ER includes chaperone-mediated assistance that can be utilized as a therapeutic targets to salvage mutant proteins from the degradation pathway (Guerriero and Brodsky, 2012). Inhibition of the ERAD pathway through pharmacological and genetic means in order to prolong the retention of mutant protein in the ER has been proven successful in a number of studies. For example, In the case of cystic fibrosis which is caused by a mutation in the cystic fibrosis transmembrane conductance regulator gene (CFTR), proteases regulators that control the folding conformation, quaternary structure of proteins have been utilized to prevent the degradation of partially folded, but functional proteins at the endoplasmic reticulum (Balch et al., 2008). In addition, it has been demonstrated that interference of the p97/VCP and GP78 ERAD complex that interacts with mutant DeltaF508-CFTR for ubiquitination and retrotranslocation out of the ER, prompted partial rescue for the DeltaF508-CFTR (Vij et al., 2006). Continued adjustment of pharmacological modulators of CFTR biogenesis have recently lead to the development of FDA approved drugs such as (Trikafta) and (Kalydeco), both used for the severely misfolded CFTR variants (Estabrooks and Brodsky, 2020). The drugs can repair CFTR misfolded variant, facilitates its escape from ERAD and promotes functionality at the cell membrane.

Lysosomal storage disorders such as Gaucher and Tay-Sachs diseases caused by mutations that affects the native folding of lysosomal enzymes are also candidates for proteasomal degradation via the ERAD mechanism (Wang et al., 2011). Research studies have shown that a combination of ERAD inhibition and upregulation of folding cellular capacity can result in mutant enzyme rescue. ERAD inhibition was achieved through small molecules named kifunensine (Kif) and Eeyarestatin I (EerI), which inhibit ER mannosidase I and p97 ATPase activities, respectively (Wang et al., 2011). Inhibition of p97/VCP has also been used for the rescue of the mutant α1 (A322D) subunit of the GABA inhibitory receptor associated with autosomal dominant juvenile myoclonic epilepsy. A combination of Eeyarestatin I and suberanilohydroxamic acid, a small molecule that enhances protein folding, restored surface expression of α1 (A322D) subunits in cell lines (Han et al., 2015). Genetic diseases such as Wilson’s disease and Progressive Familial Intrahepatic 1 cholestasis are also caused by mutations that affect the folding and the subsequent trafficking of the mutant proteins. Molecular chaperones and pharmacological means have been used to restore protein functionality (Hegde et al., 2017). More recently, we have shown that the pharmacological chaperone *N*-*n*-butyl-deoxygalactonojirimycin enhances β-galactosidase processing and activity in fibroblasts of a patient with infantile GM1-gangliosidosis (Mohamed et al., 2020). We have also proposed the modulation of mutant proteins containing the frizzled cysteine-rich domain (FZ-CRD) as a potential therapeutic target (Milhem and Ali, 2019).

Recently, ER stress has been implicated in the pathogenic mechanism of Granular corneal dystrophy type 2 (GCD2) as ER quality control mechanism delays the secretion of mutant TGFβ-induced protein (TGFβIp) through the ER/Golgi secretory pathway (Choi et al., 2016). The use of 4-PBA as therapeutic agent for GCD2 caused significant reduction in the levels of TGFβIp, BiP, and ER stress kinases in GCD2 corneal fibroblasts. These results strongly suggest that the ER quality control system plays a key role in the pathogenesis of GCD2 and proposed the chemical chaperone 4-PBA as a target therapy for this disease (Choi et al., 2016). Melatonin, which is known to suppress cell death through reduction of the UPR or ER stress, has also been used *in vitro* to relief ER stress in GCD2 corneal fibroblasts, however the exact mechanism of its action has not yet been clarified (Choi et al., 2017). Results from this study have shown reduced expression of the ERAD system components HRD1 and SEL1L, when GCD2 fibroblasts were treated with Melatonin, which makes it a potential therapeutic target for Granular corneal dystrophy type 2 (Choi et al., 2017). Genetic manipulation of the ER quality control mechanism has also been an approach adopted by many due to the latest advances in genetic editing tools. Recently, CRISPR Cas9 technology has been utilized to knockout SEL1L adaptor protein in HEK293 cell line, in order to rescue very low-density lipoprotein receptor (VLDLR) mutant protein responsible for Dysequilibrium syndrome (DES) (Ali et al., 2012). The degradation of pathogenic VLDLR and LDLR through ERAD, which is primarily dependent on SEL1-L, was considerably delayed (Kizhakkedath et al., 2018, 2019).

The above examples emphasize the potential of ERQC mechanism manipulation in the search for functional therapy for diseases with defective protein trafficking pathology. The TGFβ signaling pathway components are classic examples of secretory proteins that undergo stringent quality control checks in the ER. Therefore, TGFβ-associated genetic diseases represents potential candidates for such innovative therapy. However -up to date-restoration of defective protein trafficking in the TGFβ signaling pathway have only been investigated in few studies. In a study by Sobolewski et al. (2008), they demonstrated the potential for chemical chaperones such as thapsigargin, glycerol and sodium 4-phenylbutyrate (4-PBA) to rescue cell surface expression of mutant BMPR2 expressed in Hela cells. Restored functionality of the mutant receptor has been shown through observation of enhanced activation of SMAD1/3 signaling pathway downstream of the receptor (Sobolewski et al., 2008). In another study by Frump et al., chemical chaperones Tauroursodeoxycholic acid (TUDCA) and 4-PBA have also proven successful in partially restoring cell surface expression of mutant BMPR2 in HPAH patient-derived lymphocytes and in pulmonary endothelial cells (PECs) from HPAH mouse model (Frump et al., 2013).

Despite the small number of research studies that have been conducted for the purpose of restoring the functionality of disease-causing variants of TGFβ components, the conceptual application of such therapeutic strategy has great potentials. The examples discussed above including the usage of genetic and pharmacological means in order to prolong protein retention in the ER, lay the foundations for similar therapeutic strategies for TGFβ associated genetic diseases. Furthermore, this concept can be extended to a whole spectrum of disease-causing aberrant proteins with dysfunctional trafficking through the secretory pathway.

## Conclusion and Future Perspectives

The applicability of novel approaches to a wide range of monogenic diseases with similar pathogenic mechanisms needs to be investigated and considered as potential target for therapy. Despite progress, the full degradation pathways of mutant TGFβ component proteins such as ALK1, Endoglin, BMPR2 and other TGFβ components associated with life-limiting conditions have not yet been fully investigated. Future research should therefore focus on the elucidation of the fine details of these mechanisms in order to provide new avenue for personalized therapies. Until this date, conventional drug therapies for these diseases focus on improving the symptoms of the disease rather than modulating the molecular pathway of these aberrant proteins in the quest for restoring their functionality. Recently, however, the angiogenic molecular pathway of endothelial cells have been targeted for the treatment of HTT via inhibition of vascular endothelial growth factor (VEGF) using bevacizumab (anti-VEGF antibody) (Buscarini et al., 2019). Other preclinical studies have also identified new molecular targets that are directly linked to the signaling pathway such as (FKBP12), a protein known to interact with the BMP/TGFBRI receptor and represses its catalytic activity (PI3-kinase) downstream of VEGF and also the proangiogenic growth factor (angiopoietin-2) (Ruiz et al., 2017; Robert et al., 2020).

In this review we have shed a light on the possible implications of the ER quality control mechanisms in the pathogenesis of genetic diseases associated with mutations in components of the TGFβ signaling pathway. Therefore, therapeutic and genetic manipulation of ERAD network in order to enhance mutant protein folding and trafficking, in combination with upregulation of cellular folding capacity via molecular chaperones could be a potential strategy to rescue the localization and activity of mutant protein variants. This strategy is likely to be beneficial for biologically functional mutant proteins trapped in the ER but unable to reach their site of action due to stringent ER quality control mechanism. Hence, missense mutants of Endoglin, Alk1 and BMPR2 that fit these criteria would be an excellent target for an ERAD rescue. We believe that other TGFβ membrane and secreted components causing monogenic diseases are likely to be substrates for the ER quality control machineries and therefore further research in this area is needed. In addition, in order to make an efficient use of genetic manipulation of the ER quality control mechanism, the correlation between ERAD substrate class and the choice of the degradation channel should be further investigated in mammalian cells and animal models of disease. The realization that the location of the lesion (mutation) may determine a unique ERAD pathway, makes such a correlation essential for the characterization of the degradation pathway for ERAD substrate proteins. Despite all the progress that have been toward understanding the mechanism of ERAD machinery in mammalian cells, many questions about substrate selection and delivery to the proteasome remain unanswered. In order to utilize the full potential of ERAD as a therapeutic target for a spectrum of life-limiting disease, for which no treatment has yet been provided, these gaps are required to be filled through extensive genetic, cellular and biochemical research.

## Author Contributions

NG conducted the literature and data base searches, wrote the manuscript draft, and prepared the images and tables. BA proposed the need for writing this review, refined and edited drafts, supervised manuscript progress, and approved the final version. Both authors contributed to the article and approved the submitted version.

## Conflict of Interest

The authors declare that the research was conducted in the absence of any commercial or financial relationships that could be construed as a potential conflict of interest.
